# Supplementary material for: High-Throughput Prediction of the Band Gaps of van der Waals Heterostructures via Machine Learning
Source: Nanomaterials (Basel). 2022 Jul 4;12(13):2301. doi: 10.3390/nano12132301 (PMC9268276; doi:10.3390/nano12132301)
Supplement: Supplementary file 1 [file nanomaterials-12-02301-s001.zip › nanomaterials-1784419-supplementary.pdf]

nanomaterials

MDPI

Supplementary Materials

High-Throughput Prediction of the Band Gaps of van der Waals Heterostructures via Machine Learning

Rui Hu, Wen Lei, Hongmei Yuan, Shihao Han and Huijun Liu *

Key Laboratory of Artificial Micro- and Nano-Structures of Ministry of Education and School of Physics and Technology, Wuhan University, Wuhan 430072, China; ruihu16@whu.edu.cn (R.H.); leiwen64@whu.edu.cn (W.L.); hmyuan@whu.edu.cn (H.Y.); hansh123@whu.edu.cn (S.H.)

* Correspondence: phlhj@whu.edu.cn

Table S1. The band gaps calculated by first-principles (HSE scheme) for 325 vdWHs.

vdWHs	$E_{g,\ cal}$ (eV)	vdWHs	$E_{g,\ cal}$ (eV)	vdWHs	$E_{g,\ cal}$ (eV)
BN/BP	1.81	AlN/SnGe	0.46	GaAs/AsAs	1.58
BN/BAs	1.57	AlN/SnC	1.95	GaAs/SbSb	0.82
BN/BSb	0.98	AlN/NN	3.33	GaSb/InN	0.61
BN/AlN	3.93	AlN/PP	2.19	GaSb/InP	1.02
BN/AlP	2.88	AlN/AsAs	2.08	GaSb/InAs	0.85
BN/AlAs	2.25	AlN/SbSb	1.27	GaSb/InSb	0.75
BN/AlSb	1.99	AlP/AlAs	1.90	GaSb/SiC	1.13
BN/GaN	3.13	AlP/AlSb	1.76	GaSb/SiGe	0.22
BN/GaP	2.19	AlP/GaN	2.46	GaSb/SnSi	0.31
BN/GaAs	1.76	AlP/GaP	1.57	GaSb/GeC	1.02
BN/GaSb	1.13	AlP/GaAs	1.36	GaSb/SnGe	0.30
BN/InN	1.55	AlP/GaSb	1.09	GaSb/SnC	1.10
BN/InP	1.39	AlP/InN	1.33	GaSb/NN	1.24
BN/InAs	1.35	AlP/InP	1.15	GaSb/PP	0.96
BN/InSb	0.97	AlP/InAs	1.02	GaSb/AsAs	0.89
BN/SiC	3.27	AlP/InSb	0.84	GaSb/SbSb	0.81
BN/SiGe	0.78	AlP/SiC	2.24	InN/InP	0.60
BN/SnSi	0.63	AlP/SiGe	0.64	InN/InAs	0.43
BN/GeC	2.91	AlP/SnSi	0.59	InN/InSb	0.33
BN/SnGe	0.51	AlP/GeC	2.13	InN/SiC	1.35
BN/SnC	2.05	AlP/SnGe	0.45	InN/SiGe	0.51
BN/NN	3.89	AlP/SnC	1.79	InN/SnSi	0.55
BN/PP	2.73	AlP/NN	2.99	InN/GeC	1.15
BN/AsAs	2.06	AlP/PP	1.95	InN/SnGe	0.43
BN/SbSb	1.06	AlP/AsAs	1.96	InN/SnC	1.09
BP/BAs	1.34	AlP/SbSb	1.25	InN/NN	1.35
BP/BSb	0.91	AlAs/AlSb	1.15	InN/PP	1.30
BP/AlN	1.79	AlAs/GaN	2.35	InN/AsAs	1.25
BP/AlP	1.37	AlAs/GaP	1.06	InN/SbSb	0.82
BP/AlAs	1.29	AlAs/GaAs	1.72	InP/InAs	0.69
BP/AlSb	0.64	AlAs/GaSb	1.07	InP/InSb	0.59
BP/GaN	1.49	AlAs/InN	1.30	InP/SiC	1.16
BP/GaP	0.65	AlAs/InP	1.13	InP/SiGe	0.11

BP/GaAs	1.23	AlAs/InAs	0.90	InP/SnSi	0.30
BP/GaSb	0.59	AlAs/InSb	0.84	InP/GeC	1.06
BP/InN	1.17	AlAs/SiC	2.23	InP/SnGe	0.19
BP/InP	0.52	AlAs/SiGe	0.63	InP/SnC	0.96
BP/InAs	0.35	AlAs/SnSi	0.57	InP/NN	1.06
BP/InSb	0.25	AlAs/GeC	2.02	InP/PP	0.84
BP/SiC	1.59	AlAs/SnGe	0.44	InP/AsAs	0.75
BP/SiGe	0.66	AlAs/SnC	1.62	InP/SbSb	0.81
BP/SnSi	0.61	AlAs/NN	2.35	InAs/InSb	0.97
BP/GeC	0.38	AlAs/PP	1.77	InAs/SiC	1.01
BP/SnGe	0.48	AlAs/AsAs	2.01	InAs/SiGe	0.11
BP/SnC	1.01	AlAs/SbSb	1.22	InAs/SnSi	0.13
BP/NN	1.69	AlSb/GaN	1.58	InAs/GeC	0.98
BP/PP	1.43	AlSb/GaP	1.50	InAs/SnGe	0.10
BP/AsAs	1.09	AlSb/GaAs	0.92	InAs/SnC	0.94
BP/SbSb	0.64	AlSb/GaSb	0.88	InAs/NN	1.04
BAs/BSb	0.90	AlSb/InN	0.72	InAs/PP	0.28
BAs/AlN	1.43	AlSb/InP	1.12	InAs/AsAs	0.71
BAs/AlP	1.03	AlSb/InAs	1.00	InAs/SbSb	0.83
BAs/AlAs	0.93	AlSb/InSb	0.82	InSb/SiC	0.87
BAs/AlSb	0.60	AlSb/SiC	1.89	InSb/SiGe	0.05
BAs/GaN	1.28	AlSb/SiGe	0.23	InSb/SnSi	0.04
BAs/GaP	0.51	AlSb/SnSi	0.32	InSb/GeC	0.92
BAs/GaAs	0.94	AlSb/GeC	2.04	InSb/SnGe	0.08
BAs/GaSb	0.59	AlSb/SnGe	0.31	InSb/SnC	1.01
BAs/InN	1.23	AlSb/SnC	1.38	InSb/NN	0.97
BAs/InP	0.58	AlSb/NN	1.71	InSb/PP	0.46
BAs/InAs	0.31	AlSb/PP	0.67	InSb/AsAs	0.60
BAs/InSb	0.25	AlSb/AsAs	0.90	InSb/SbSb	0.80
BAs/SiC	1.45	AlSb/SbSb	1.12	SiC/SiGe	0.58
BAs/SiGe	0.65	GaN/GaP	1.49	SiC/SnSi	0.62
BAs/SnSi	0.59	GaN/GaAs	1.55	SiC/GeC	2.51
BAs/GeC	1.43	GaN/GaSb	1.06	SiC/SnGe	0.46
BAs/SnGe	0.56	GaN/InN	1.54	SiC/SnC	1.85
BAs/SnC	1.07	GaN/InP	1.14	SiC/NN	2.79
BAs/NN	1.43	GaN/InAs	1.02	SiC/PP	2.09
BAs/PP	1.31	GaN/InSb	0.93	SiC/AsAs	1.55
BAs/AsAs	1.03	GaN/SiC	2.70	SiC/SbSb	1.27
BAs/SbSb	0.70	GaN/SiGe	1.22	SiGe/SnSi	0.50
BSb/AlN	0.91	GaN/SnSi	0.78	SiGe/GeC	0.82
BSb/AlP	0.71	GaN/GeC	2.14	SiGe/SnGe	0.73
BSb/AlAs	0.70	GaN/SnGe	0.45	SiGe/SnC	0.55
BSb/AlSb	0.46	GaN/SnC	1.87	SiGe/NN	0.98
BSb/GaN	0.88	GaN/NN	2.35	SiGe/PP	0.85
BSb/GaP	0.37	GaN/PP	1.88	SiGe/AsAs	0.65
BSb/GaAs	0.63	GaN/AsAs	2.04	SiGe/SbSb	0.23
BSb/GaSb	0.45	GaN/SbSb	1.24	SnSi/GeC	0.59
BSb/InN	0.61	GaP/GaAs	0.81	SnSi/SnGe	0.52
BSb/InP	0.44	GaP/GaSb	0.75	SnSi/SnC	0.49
BSb/InAs	0.27	GaP/InN	0.73	SnSi/NN	0.79
BSb/InSb	0.17	GaP/InP	0.79	SnSi/PP	0.53

BSb/SiC	0.95	GaP/InAs	1.01	SnSi/AsAs	0.51
BSb/SiGe	0.52	GaP/InSb	0.81	SnSi/SbSb	0.42
BSb/SnSi	0.58	GaP/SiC	1.90	GeC/SnGe	0.44
BSb/GeC	0.92	GaP/SiGe	0.14	GeC/SnC	1.85
BSb/SnGe	0.45	GaP/SnSi	0.23	GeC/NN	2.58
BSb/SnC	0.81	GaP/GeC	1.85	GeC/PP	1.39
BSb/NN	1.01	GaP/SnGe	0.22	GeC/AsAs	1.77
BSb/PP	0.97	GaP/SnC	1.29	GeC/SbSb	1.23
BSb/AsAs	0.61	GaP/NN	1.62	SnGe/SnC	0.46
BSb/SbSb	0.56	GaP/PP	0.58	SnGe/NN	0.47
AlN/AlP	2.94	GaP/AsAs	0.81	SnGe/PP	0.45
AlN/AlAs	2.35	GaP/SbSb	1.03	SnGe/AsAs	0.42
AlN/AlSb	2.04	GaAs/GaSb	0.61	SnGe/SbSb	0.39
AlN/GaN	3.20	GaAs/InN	1.25	SnC/NN	1.85
AlN/GaP	2.33	GaAs/InP	0.60	SnC/PP	1.04
AlN/GaAs	1.60	GaAs/InAs	0.42	SnC/AsAs	1.37
AlN/GaSb	1.11	GaAs/InSb	0.33	SnC/SbSb	1.20
AlN/InN	2.35	GaAs/SiC	1.70	NN/PP	2.73
AlN/InP	1.27	GaAs/SiGe	0.52	NN/AsAs	1.96
AlN/InAs	1.14	GaAs/SnSi	0.56	NN/SbSb	1.28
AlN/InSb	0.95	GaAs/GeC	1.49	PP/AsAs	1.53
AlN/SiC	3.07	GaAs/SnGe	0.42	PP/SbSb	0.87
AlN/SiGe	0.86	GaAs/SnC	1.10	AsAs/SbSb	0.68
AlN/SnSi	0.70	GaAs/NN	1.88		
AlN/GeC	2.51	GaAs/PP	1.73		
